# Optimal capacity configuration of wind-photovoltaic-storage hybrid systems based on improved chaotic evolution optimization algorithm

**DOI:** 10.1038/s41598-026-40610-7

**Published:** 2026-02-20

**Authors:** Yingchao Dong, Xiang Zhou, Xiguo Cao, Jiading Jiang, Yan He, Cui Yin

**Affiliations:** 1 School of Energy Engineering, Xinjiang Institute of Engineering, Urumqi, 830023 China; 2 School of Information Management, Xinjiang University of Finance and Economics, Urumqi, 830012 China

**Keywords:** Wind–photovoltaic–storage system, Capacity configuration, Chaotic evolution optimization, Self-learning perturbation, Adaptive local search, Energy science and technology, Engineering, Mathematics and computing

## Abstract

This study addresses the optimal capacity configuration of wind–photovoltaic–storage (WPS) systems under complex nonlinear constraints and economic requirements in grids with a high share of renewable energy. A multi-energy collaborative capacity planning model is developed, together with an energy management formulation that captures the coupling among wind, PV, and storage. To solve the resulting constrained optimization problem, an improved chaotic evolution optimization algorithm (ICEO) is proposed by embedding a self-learning perturbation strategy and an adaptive local search mechanism into the chaotic evolution framework. Specifically, Gaussian mutation and Lévy flight are combined to generate cooperative perturbations around high-quality solutions, while a stagnation-triggered local search refines solutions when the population evolution slows down. Simulation results on standard benchmark functions and a practical WPS case study demonstrate that ICEO achieves higher solution quality and robustness than several state-of-the-art meta-heuristics, thereby improving cost-effectiveness for WPS capacity planning.

## Introduction

With the increasing penetration of wind and photovoltaic generation driven by carbon-neutrality targets, power systems face pronounced stochasticity and uncertainty, which erode operating margins and threaten system stability^1^. Therefore, coordinated capacity configuration of energy storage systems (ESS) is essential to simultaneously improve economic performance, reliability, and renewable energy utilization, thereby enhancing the stability of grid-integrated renewable systems^2^.

Regarding the optimal capacity allocation problem in wind–photovoltaic–storage (WPS) systems, extensive studies have been reported. Reference^3^ proposes a multi-energy flow power supply system integrating wind power, PV, ESS, and fuel cells, and achieves optimal configuration of equipment capacity through an improved genetic algorithm (GA). Reference^4^ proposes a hybrid PSO–NSGA-II method for the capacity design of a wind/photovoltaic/hydrogen storage system, achieving an 87.38% constraint satisfaction rate, and reducing economic cost by 6.3 million CNY. Reference^5^ proposes an improved multi-objective salp swarm algorithm for capacity optimization of a wind–PV–hydrogen system, incorporating tent chaotic mapping and an adaptive spiral search strategy, which reduces annual planning cost and power supply loss rate. Reference^6^ establishes a hybrid ESS model including wind power, PV, lithium ESS, and hydrogen ESS, and solves the configuration using an inertia-weight dynamically adjusted PSO. Reference^7^ proposes a capacity optimization method for distributed WPS systems based on scenario generation, using Latin hypercube sampling to generate wind/solar scenarios and fast backward reduction to obtain typical scenarios, and then maximizing system net revenue with storage capacity as the decision variable. Reference^8^ employs the sparrow search algorithm to solve a capacity configuration optimization model aimed at minimizing total costs, providing an effective approach for planning WPS hybrid systems. In addition, recent studies have investigated ESS sizing for smoothing renewable fluctuations^9–11^, multiobjective capacity planning for multi-energy complementary systems^12–14^, and WPS scheduling considering battery service life^15^.

Despite these advances, WPS planning remains challenging due to strongly nonlinear constraints, time-coupled operational limits, and rugged fitness landscapes that can induce premature convergence of meta-heuristics. In particular, robust feasibility handling and search efficiency under such constraints remain open challenges. Moreover, reliable capacity planning depends on accurate modeling of renewable generation and ESS dynamics. These challenges motivate the development of more robust optimization frameworks, such as CEO^16^, which is a chaos-driven evolutionary framework that adopts the mutation–crossover–selection procedure commonly used in differential evolution. Its performance is typically assessed on benchmark problems^17^ and compared with representative meta-heuristics (e.g., PSO^18^ and INFO^19^). Table 1 summarizes selected representative studies and positions the proposed ICEO within this literature.

**Table 1:** Representative studies on WPS capacity optimization.

Reference	System Type	Objectives	Algorithms
^3^	WPS–fuel cell	Equipment cost	Improved GA
^4^	Wind/PV/Hydrogen storage	Capacity design under constraints	PSO–NSGA-II
^5^	Wind–PV–Hydrogen–Battery	Total annual planning cost, lowest self-supporting loss rate	Improved multi-objective salp swarm algorithm
^6^	Wind–PV–Lithium–hydrogen storage	Investment and operation cost, suppress fluctuation	Inertia-weight dynamically adjusted PSO
^7^	Distributed WPS	Net revenue under uncertainty	Scenario generation + Cplex
^8^	WPS systems	Investment and operation cost	Sparrow search algorithm
**This paper**	**WPS systems**	**Min. total installed cost**	**ICEO**

Addressing this limitation, this paper proposes an ICEO algorithm for WPS capacity optimization configuration. The primary contribution of this work lies in the tailored integration of self-learning and local search mechanisms into the chaotic evolution framework, specifically designed to address the rugged fitness landscapes and intricate coupling common in WPS capacity planning. Unlike generic improvements, the ICEO’s self-learning strategy adaptively scales the search step based on the current best individual, which is particularly effective for fine-tuning the sensitive balance between wind, solar, and storage capacities. By incorporating Gaussian mutation and Lévy flight self-learning strategies, along with an adaptive local search mechanism based on stagnation detection, the algorithm dynamically adjusts mutation intensity to enhance global exploration capabilities and local exploitation precision. Finally, the ICEO algorithm is applied to a typical WPS capacity optimization problem and compared with several other advanced intelligent optimization methods, demonstrating its effectiveness and advantages of the proposed algorithm.

## Mathematical model of WPS power output

### Wind power generation output model

A common model for the electrical power generation $P_\text {{WT}}$ of a wind turbine as a function of wind speed $v$ is given by the piecewise-defined power curve:

1 $$\begin{aligned} P_\text {WT} = {\left\{ \begin{array}{ll} 0 & \text {if } v \le v_{\text {in}} \text { or } v \ge v_{\text {out}} \\ P_{\text {WT}}^{\text {cap}} \dfrac{v^3 - v_{\text {in}}^3}{v_N^3 - v_{\text {in}}^3} & \text {if } v_{\text {in}} \le v \le v_N \\ P_{\text {WT}}^{\text {cap}} & \text {if } v_N \le v \le v_{\text {out}} \end{array}\right. } \end{aligned}$$

where $P_\text {WT}$ and $P_\text {WT}^\text {cap}$ are the actual output power and rated installed capacity of the wind turbine, respectively; $v$, $v_N$, $v_{\text {in}}$ and $v_{\text {out}}$ are the actual wind speed, rated wind speed, cut-in wind speed, and cut-out wind speed, respectively.

### PV power generation output model

The output power of PV modules is primarily determined by the rated capacity, solar irradiance, and cell temperature. These quantities are influenced by installation conditions (e.g., module technology, installation area, solar resource level, and electrical interconnection)^20^. The PV output model can be expressed as:

2 $$\begin{aligned} P_\text {{PV}} = P_{\text {PV}}^{\text {cap}} \times \frac{G_\text {PV}}{G_{\text {stc}}} \times \left( 1 + \alpha \times (T_\text {PV} - T_{\text {stc}}) \right) \end{aligned}$$

where $P_\text {PV}$ and $P_\text {PV}^\text {cap}$ are the real-time output power and rated installed capacity of the PV array, respectively; $G_\text {PV}$ and $G_{\text {stc}}$ denote the actual irradiance and the irradiance under standard test conditions, respectively; $\alpha$ is the power temperature coefficient; and $T_\text {PV}$ and $T_{\text {stc}}$ are the PV operating temperature and the reference temperature under standard test conditions, respectively.

### Energy storage model

In the charging and discharging process of the ESS, charging and discharging cannot occur simultaneously. The state of charge (SOC) is updated as follows:

3 $$\begin{aligned} \text {SOC}(t) = \text {SOC}(t-1) + \eta _{\text {ch}} \frac{P_{\text {ESS}}^{\text {ch}} \Delta t}{E_{\text {ESS}}^{\text {cap}}} - \frac{P_{\text {ESS}}^{\text {dis}} \Delta t}{\eta _{\text {dis}} E_{\text {ESS}}^{\text {cap}}} \end{aligned}$$

where $\text {SOC}(t)$ and $\text {SOC}(t-1)$ denote the SOC of the ESS at time $t$ and the previous time step $t-1$, respectively; $\eta _{\text {ch}}$ and $\eta _{\text {dis}}$ represent the charging and discharging efficiencies of the ESS, respectively; $P_{\text {ESS}}^{\text {dis}}$ and $P_{\text {ESS}}^{\text {ch}}$ represent the ESS discharging and charging powers, respectively; $\Delta t$ denotes the time step; and $E_{\text {ESS}}^{\text {cap}}$ denotes the rated energy capacity of the ESS.

## Capacity configuration optimization model

### Objective function

To minimize the total installed cost of wind turbines, PV modules, and ESS, a collaborative multi-variable capacity configuration model is formulated. The installed capacities of wind, PV, and storage are treated as continuous decision variables, subject to operational constraints including time-coupled power balance and ESS state-of-charge limits. The objective incorporates both investment and operation and maintenance (O&M) costs, as well as the cost of power exchange with the main grid.

4 $$\begin{aligned} C_{\text {total}} = \min (C_\text {{WT}} + C_\text {{PV}} + C_\text {{ESS}} + C_{\text {grid}}) \end{aligned}$$

where $C_{\textrm{total}}$ denotes the total installed capacity cost of the system, and $C_{\textrm{WT}}$, $C_{\textrm{PV}}$, $C_{\textrm{ESS}}$, and $C_{\textrm{grid}}$ represent the costs associated with wind turbines, photovoltaic modules, energy storage systems, and transactions with the external grid, respectively.

5 $$\begin{aligned} {\left\{ \begin{array}{ll} C_{WT} = c_\text {{WT}}^{\text {inv}} \frac{\gamma (1+\gamma )^m}{(1+\gamma )^m - 1} P_{\text {WT}}^{\text {cap}} + c_\text {{WT}}^{\text {om}} P_{\text {WT}}^{\text {cap}} \\ C_{PV} = c_\text {{PV}}^{\text {inv}} \frac{\gamma (1+\gamma )^m}{(1+\gamma )^m - 1} P_{\text {PV}}^{\text {cap}} + c_\text {{PV}}^{\text {om}} P_{\text {PV}}^{\text {cap}} \\ C_{ESS} = c_\text {{ESS}}^{\text {inv}} \frac{\gamma (1+\gamma )^m}{(1+\gamma )^m - 1} E_{\text {ESS}}^{\text {cap}} + c_\text {{ESS}}^{\text {om}} E_{\text {ESS}}^{\text {cap}}\\ C_{\textrm{buy}} = 365 \times \sum _{t=1}^{T} c_{\textrm{ele}}(t) \, P_{\textrm{grid}}(t) \end{array}\right. } \end{aligned}$$

where $c_{\textrm{WT}}^{\textrm{inv}}$, $c_{\textrm{PV}}^{\textrm{inv}}$, and $c_{\textrm{ESS}}^{\textrm{inv}}$ denote the unit capacity investment costs for wind power, PV, and energy storage equipment, respectively; $C_{\textrm{WT}}^{\textrm{om}}$, $C_{\textrm{PV}}^{\textrm{om}}$, and $C_{\textrm{ESS}}^{\textrm{om}}$ represent the fixed operation and maintenance costs per unit capacity for the corresponding equipment; $\gamma$ is the depreciation rate of the equipment; $m$ denotes the service life of the equipment; while $c_{\textrm{ele}}(t)$ and $P_{\textrm{grid}}(t)$ refer to the electricity transaction price and transacted power with the grid at time $t$, respectively.

### Constraint conditions

#### Power balance constraint

6 $$\begin{aligned} P_{\text {PV}}(t) + P_{\text {WT}}(t) + P_{\text {ESS}}^{\text {dis}}(t) - P_{\text {ESS}}^{\text {ch}}(t) = P_{\text {load}}(t) + P_{\text {grid}}(t) \end{aligned}$$

where $P_{\text {PV}}(t)$ denotes the PV output power at time $t$; $P_{\text {WT}}(t)$ denotes the wind turbine output power at time $t$; $P_{\text {load}}(t)$ denotes the load demand at time $t$; and $P_{\text {ESS}}^{\text {ch}}(t)$ and $P_{\text {ESS}}^{\text {dis}}(t)$ represent the charging and discharging powers of the ESS at time $t$, respectively.

#### Capacity range constraint

The installed capacities of wind, PV, and ESS are bounded as follows:

7 $$\begin{aligned} {\left\{ \begin{array}{ll} 0 \le P_{\text {WT}}^{\text {cap}} \le P_{\text {WT}}^{\text {max}} \\ 0 \le P_{\text {PV}}^{\text {cap}} \le P_{\text {PV}}^{\text {max}} \\ 0 \le E_{\text {ESS}}^{\text {cap}} \le E_{\text {ESS}}^{\text {max}} \end{array}\right. } \end{aligned}$$

where $P_\text {{WT}}^{\max }$, $P_\text {{PV}}^{\max }$, $E_\text {{ESS}}^{\max }$ represent the maximum installed capacity of wind power, PV power, and energy storage, respectively.

#### ESS constraints

The SOC is defined as the ratio of the available energy to the rated energy capacity of the ESS, reflecting the remaining stored energy^21^. The SOC constraint is formulated as:

8 $$\begin{aligned} \text {SOC}_{\text {min}} \le \text {SOC}(t) \le \text {SOC}_{\text {max}} \end{aligned}$$

where $\text {SOC}_{\text {min}}$ and $\text {SOC}_{\text {max}}$ are the lower and upper limits of the ESS SOC, respectively; $\text {SOC}(t)$ represents the SOC of the ESS at time $t$.

9 $$\begin{aligned} {\left\{ \begin{array}{ll} 0 \le P_{\text {ESS}}^{\text {ch}}(t) \le P_{\text {ESS}} \\ 0 \le P_{\text {ESS}}^{\text {dis}}(t) \le P_{\text {ESS}} \\ P_{\text {ESS}}^{\text {ch}}(t) \cdot P_{\text {ESS}}^{\text {dis}}(t) = 0 \end{array}\right. } \end{aligned}$$

where $P_{\text {ESS}}$ denotes the rated power of the ESS.

#### Tie-line Power Constraint

10 $$\begin{aligned} -P_{\text {grid}}^{\text {max}} \le P_{\text {grid}}(t) \le P_{\text {grid}}^{\text {max}} \end{aligned}$$

where $P_{\text {grid}}^{\text {max}}$ denotes the power exchange limit with the main grid. A positive value $P_{\text {grid}}(t) > 0$ indicates electricity purchase from the grid, whereas $P_{\text {grid}}(t) < 0$ indicates electricity export to the grid.

## ICEO algorithm

### Algorithm framework

To solve the WPS capacity configuration model, this paper proposes an improved chaotic evolution optimization algorithm (ICEO) developed from chaotic evolution optimization (CEO)^16^. CEO is inspired by chaotic dynamics and uses the hyperchaotic mapping of a two-dimensional discrete memristor system to generate multiple search directions and guide population evolution. Compared with conventional one-dimensional chaotic maps, the hyperchaotic map incorporates inter-individual interactions and memory effects, thereby enhancing diversity and facilitating exploration of promising regions. CEO also follows the mutation–crossover–selection procedure of differential evolution (DE)^22^. Although CEO can be viewed as a DE-type framework, it differs from canonical DE in the following aspects:

*(1) Mutation operator*

The mutation operator in CEO differs from that in DE. CEO utilizes sequences generated by memristor-based hyperchaotic mapping to guide population evolution, providing more randomized search directions compared to DE. This helps avoid the issue where difference terms approach zero in the later stages of DE evolution, which can lead to local optima traps or stagnation.

*(2) Evolutionary directions*

In DE, each individual has only one evolutionary direction. However, in CEO, each individual can generate multiple chaotic search directions. This enhancement significantly improves the algorithm’s ability to explore and utilize current individuals, thereby increasing the probability of finding the global optimal solution.

*(3) Crossover control parameter*

In DE, the crossover control parameter $Cr$ is typically fixed within $[0,1]$. In contrast, CEO samples $Cr$ randomly from $[0,1]$ at each iteration. Moreover, the fixed scaling factor $F$ in DE is replaced by a random number uniformly distributed in $[0,1]$. These modifications increase stochasticity in variation operators, improve population diversity, and mitigate premature convergence to local optima, thereby enhancing the robustness of the search process.

In ICEO, a self-learning perturbation strategy and an adaptive local search mechanism are embedded into the CEO framework to improve the capability of escaping local optima. Specifically, when the currently selected individual coincides with the global best solution, Gaussian self-learning mutation and Lévy flight perturbation are applied to generate multiple candidate trial solutions around the best solution. Furthermore, a stagnation detection mechanism is introduced to trigger a local search (LS) refinement when the population evolution slows down, thereby enhancing both exploration and exploitation in complex constrained search spaces.

The distinct contribution of ICEO to WPS optimization, compared to the original CEO, lies in its ability to navigate the highly non-linear and rugged cost landscapes of energy systems. While CEO provides a strong foundation through chaotic sampling, it can still struggle with the sensitive balance between different energy components (wind, solar, and storage) where the optimal regions are often narrow and surrounded by numerous local optima. The self-learning perturbation allows ICEO to explore the vicinity of the global best solution with varying intensities–Gaussian mutation for fine-tuning and Lévy flight for escaping deep local optima. The adaptive local search further ensures that once a promising region is identified, the algorithm can rapidly converge to the high-precision solution required for economic planning. This multi-strategy integration is specifically tailored to the intricate coupling and sensitive constraints inherent in WPS capacity configuration.

### Chaotic evolution

*(1) Mutation operator*

Initialize parameters including the number of chaotic sampling points $N$, population size $Np$. Create an initial population, calculate the fitness of the initial population, and determine the initial optimal solution $Best$ and the optimal function value $fBest$. Select two individuals $x_i$ and $y_i$, and map them to the intervals $[-0.5, 0.5]$ and $[-0.25, 0.25]$, respectively; ensuring they reside within the hyperchaotic attractor domain.

11 $$\begin{aligned} {\left\{ \begin{array}{ll} x_i' = \dfrac{x_i - lb}{ub - lb} - 0.5 \\ y_i' = \dfrac{y_i - lb}{ub - lb} \times 0.5 - 0.25 \end{array}\right. } \end{aligned}$$

where $x_i'$ and $y_i'$ are the chaotic initial positions after mapping; their values are located within the intervals $[-0.5, 0.5]$ and $[-0.25, 0.25]$, respectively; $lb$ and $ub$ are the lower and upper bounds of the current population variables.

*(2)Chaotic sampling*

For $n = 1, 2, \ldots , N$, the hyperchaotic map is iterated as:

12 $$\begin{aligned} {\left\{ \begin{array}{ll} x\_chaos(n,:) = k \cdot \left( e^{-\cos \pi y_t'} - 1 \right) \cdot x_t' \\ y\_chaos(n,:) = y_t' + x_t' \end{array}\right. } \end{aligned}$$

where the number of chaotic sampling points is set to *N* to guide two individuals simultaneously as per the memristor-based hyperchaotic mapping framework. The control parameter $k=2.66$ is selected to ensure the chaotic mapping remains within the chaotic attractor region for effective randomized search.

*(3)Inverse mapping to obtain the actual location*

13 $$\begin{aligned} {\left\{ \begin{array}{ll} x\_chaos^{n'} = (x\_chaos^n + 0.5) \times (ub - lb) + lb \\ y\_chaos^{n'} = (y\_chaos^n + 0.25) \times 2 \times (ub - lb) + lb \end{array}\right. } \end{aligned}$$

where $x\_chaos^{n'}$ and $y\_chaos^{n'}$ denote the inverse-mapped chaotic samples in the original decision space, which are used to construct $N$ candidate search directions for $x_t$ and $y_t$, respectively.

*(4)Evolutionary direction *

14 $$\begin{aligned} {\left\{ \begin{array}{ll} d_{x,t}^n = x\_chaos^{n'} - x_t \\ d_{y,t}^n = y\_chaos^{n'} - y_t \end{array}\right. } \end{aligned}$$

where $d_{x,t}^n$ and $d_{y,t}^n$ represent the n-th evolutionary direction of individual $x_t$ and $y_t$, respectively.

### Mutation and crossover

#### Mutation operation

*(1)Current position mutation:*

15 $$\begin{aligned} {\left\{ \begin{array}{ll} \tilde{x}_{t+1}^n = x_t + a \cdot (x\_chaos^{n'} - x_t) \\ \tilde{y}_{t+1}^n = y_t + a \cdot (y\_chaos^{n'} - y_t) \end{array}\right. } \end{aligned}$$

where $\tilde{x}_{t+1}^n$ and $\tilde{y}_{t+1}^n$ are the new individual position vectors for $x_t$ and $y_t$; $a$ is the dynamic mutation step size, which is randomized within [0, 1] at each iteration to enhance stochastic exploration and prevent early convergence.

*(2)Best position mutation:*

16 $$\begin{aligned} {\left\{ \begin{array}{ll} \tilde{x}_{t+1}^n = Best_t + a \cdot (x\_chaos^{n'} - x_t) \\ \tilde{y}_{t+1}^n = Best_t + a \cdot (y\_chaos^{n'} - y_t) \end{array}\right. } \end{aligned}$$

where $Best_t$ is the optimal individual position in the population.

#### Crossover operation

For the mutated individuals $(x_t, \tilde{x}_{t+1}^n)$ and $(y_t, \tilde{y}_{t+1}^n)$, apply binomial crossover to generate trial vectors:

17 $$\begin{aligned} x_{trial_{j,t}}^n = {\left\{ \begin{array}{ll} \tilde{x}_{j,t+1}^n & \text {if } (rand_j(0,1] \le C_r) \text { or } (j = j_{rand}) \\ x_{j,t} & \text {otherwise} \end{array}\right. } \end{aligned}$$

where $x_{trial_{j,t}}^n$ denotes the $n$-th trial vector generated by the binomial crossover operation; $\tilde{x}_{j,t+1}^n$ denotes the $n$-th mutated vector obtained after the mutation operation; $x_{j,t}$ denotes the original vector in the $j$-th dimension at time $t$; and $Cr$ is the crossover control parameter, randomized within [0, 1] to introduce adaptive diversity in the decision variables (e.g., wind, solar, and storage capacities).

18 $$\begin{aligned} y_{trial_{j,t}}^n = {\left\{ \begin{array}{ll} \tilde{y}_{j,t+1}^n & \text {if } (rand_j(0,1] \le C_r) \text { or } (j = j_{rand}) \\ y_{j,t} & \text {otherwise} \end{array}\right. } \end{aligned}$$

where $y_{trial_{j,t}}^n$ represents the trial vector generated through the binomial crossover operation; $\tilde{y}_{j,t+1}^n$ represents the $n$-th new individual vector obtained after the mutation operation; $y_{j,t}$ represents the original individual vector in the $j$-th dimension at time $t$.

### Greedy selection

19 $$\begin{aligned} {\left\{ \begin{array}{ll} x_{t+1} = {\left\{ \begin{array}{ll} x\_trial_t^* & \text {if } f(x\_trial_t^*) \le f(x_t) \\ {x_t} & \text {otherwise} \end{array}\right. } \\ y_{t+1} = {\left\{ \begin{array}{ll} y\_trial_t^* & \text {if } f(y\_trial_t^*) \le f(y_t) \\ {y_t} & \text {otherwise} \end{array}\right. } \end{array}\right. } \end{aligned}$$

where $x_{t+1}$ and $y_{t+1}$ represent the new individuals determined after selection according to the greedy criterion; $x\_trial_t^*$ and $y\_trial_t^*$ represent the trial vectors that are selected for comparison with the current individuals $x_t$ and $y_t$ after evaluation among all the generated trial vectors $x\_trial_t^n$ and $y\_trial_t^n$.

### Self-learning strategy

The self-learning strategy in ICEO consists of two components: Gaussian mutation and Lévy flight. In particular, these two operators are activated when generating trial solutions around the current global best solution, forming a self-adaptive perturbation mechanism that strengthens the ability to escape local optima while maintaining fast convergence.

*(1)Gaussian mutation*

In ICEO, a Gaussian perturbation term is applied to the current-best solution. The best solution is perturbed by random numbers drawn from a standard normal distribution to form multiple candidate solutions, and the perturbation amplitude is proportional to the best solution itself, thereby reflecting the self-learning characteristic.

20 $$\begin{aligned} Best' = Best + r_1 \cdot Best \end{aligned}$$

where $Best'$ is the candidate solution obtained through Gaussian self-learning; $Best$ is the best solution for the current iteration; $r_1$ follows the standard normal distribution.

*(2)Lévy flight*

To further perturb the best solution, a Lévy flight operation is applied. The perturbation utilizes a random step size drawn from a Lévy distribution with heavy tails, which helps the algorithm perform occasional long-distance jumps to escape local optima and enhance global exploration. The step length of the Lévy flight is generated as follows:

21 $$\begin{aligned} S = \frac{u}{|\mu |^{1/\beta }} \end{aligned}$$

where $u$ and $\mu$ are random variables following a normal distribution, that is:

22 $$\begin{aligned} {\left\{ \begin{array}{ll} u \sim N(0, \sigma _u^2) \\ \mu \sim N(0, \sigma _\mu ^2) \end{array}\right. } \end{aligned}$$

The standard deviations of $\sigma _u$ and $\sigma _\mu$ are calculated as:

23 $$\begin{aligned} {\left\{ \begin{array}{ll} \sigma _u = \left( \dfrac{\Gamma (1 + \beta ) \cdot \sin \frac{\pi \beta }{2}}{\Gamma \left( \dfrac{1 + \beta }{2} \right) \cdot \beta \cdot 2^{\frac{\beta - 1}{2}}} \right) ^{\frac{1}{\beta }} \\ \sigma _\mu = 1 \end{array}\right. } \end{aligned}$$

where $\beta$ is a parameter of the Lévy distribution, usually taking values in the range of $(0, 2]$. In this work, $\beta =1.5$ is selected as it provides a robust balance between short-range exploitation and long-range exploration, facilitating the escape from local optima in the rugged cost landscape of the WPS system. $u$ and $\mu$ are two independent normally distributed random variables, used to generate the Lévy step length. $\Gamma$ is a normalization coefficient for calculating $\sigma _u$, ensuring that the statistical characteristics of the generated step length are consistent with the theoretical Lévy distribution.

To sum up, based on the best solution of the current iteration, the combined self-learning search strategy with Gaussian mutation and Lévy flight can be expressed as:

24 $$\begin{aligned} Best' = Best + r_1 \cdot Best + r_2 \cdot Levy(\beta ) \end{aligned}$$

where $r_2$ is a random number uniformly distributed in the interval $[0, 1]$.

### Stagnation detection and LS local search

To improve solution quality when the population evolution becomes stagnant, ICEO introduces an LS (local search) refinement stage on the current best solution. After each generation, the algorithm evaluates a stagnation indicator based on the relative change of the population mean fitness. Let $\bar{f}^{(t)}$ denote the mean fitness of the population at generation $t$. The stagnation test is defined as:

25 $$\begin{aligned} \rho ^{(t)}=\left| \frac{\bar{f}^{(t-1)}-\bar{f}^{(t)}}{\bar{f}^{(t-1)}}\right| \end{aligned}$$

If $\rho ^{(t)} < \rho _{th}$, the evolution is regarded as stagnant and LS is triggered immediately. In this study, the stagnation threshold is set to $\rho _{th}=10^{-3}$. This mechanism corresponds to monitoring $\bar{f}^{(t)}$ computed from the population fitness values and invoking LS once the relative improvement becomes sufficiently small. The LS call can be written as:

26 $$\begin{aligned} \left( Best^{LS}, fBest^{LS}, \Delta FE_{LS}\right) =LS\!\left( Best, fBest, lb, ub, f(\cdot )\right) \end{aligned}$$

where $lb$ and $ub$ are the lower and upper bounds, respectively; $Best^{LS}$ and $fBest^{LS}$ are the refined solution and its objective value returned by LS; and $\Delta FE_{LS}$ denotes the additional function evaluations consumed by LS, which are accumulated into the overall evaluation budget. If $fBest^{LS} < fBest$, the current best solution is updated by $Best \leftarrow Best^{LS}$ and $fBest \leftarrow fBest^{LS}$. In practice, LS is implemented via a constrained local optimizer (SQP-type), but the specific solver details are omitted here for brevity.

### Flowchart of algorithm

**Fig. 1:**
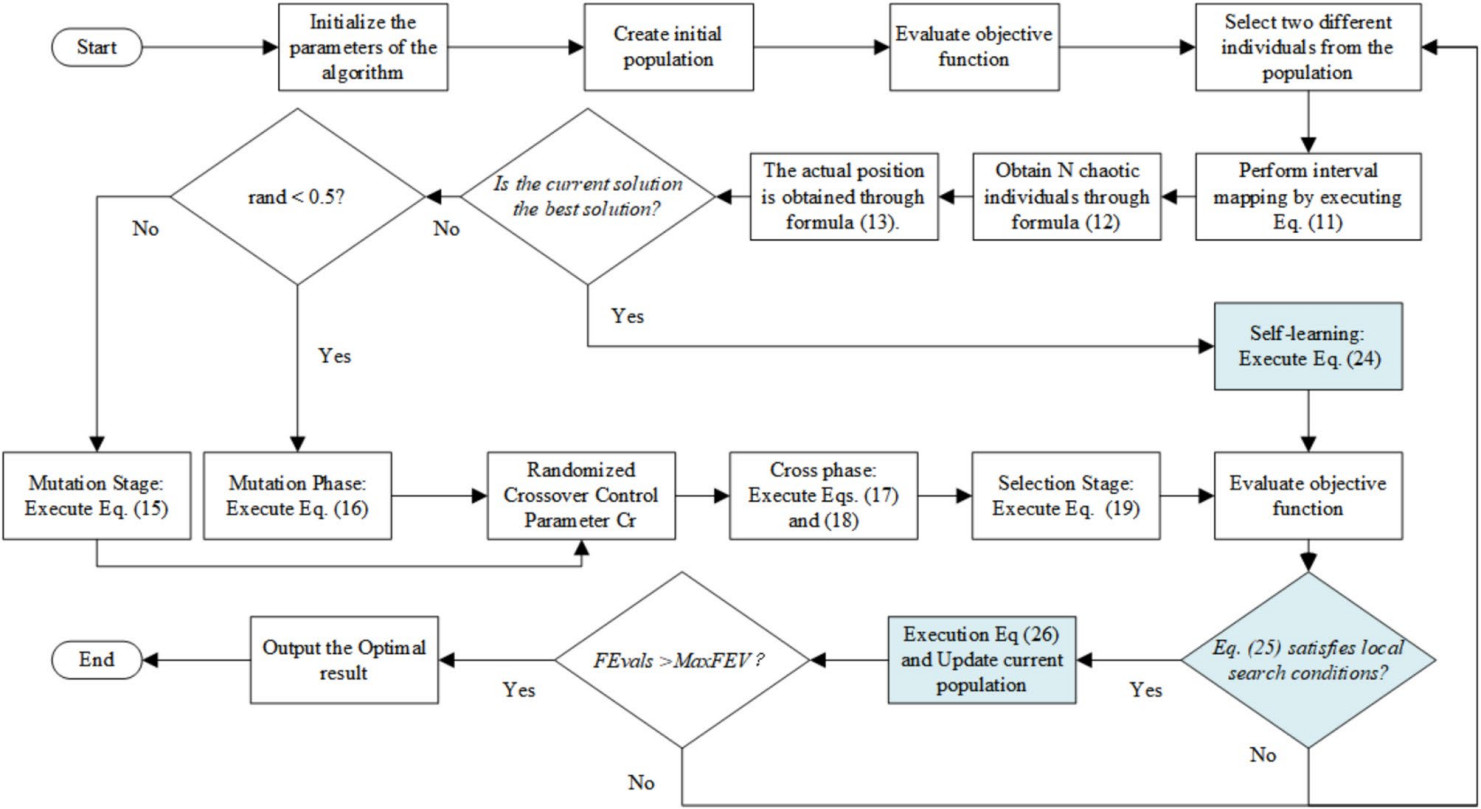
Flowchart of the ICEO algorithm.

Figure 1 illustrates the detailed execution flow of the proposed ICEO. Compared with the basic CEO framework, the flow additionally includes the stagnation detection step and the optional LS local search refinement on the current best solution.

### Benchmark test of ICEO algorithm

To verify the optimization performance of the proposed ICEO algorithm, experiments are conducted on 8 standard benchmark functions^17^, including 4 unimodal functions (Sphere, Schwefel 2.22, Tablet, Zakharov) and 4 multimodal functions (Ackley, Griewank, Rastrigin, Schwefel 2.26). The unimodal set (F1–F4) is used to evaluate convergence behavior and exploitation precision, whereas the multimodal set (F5–F8) is used to test global exploration and the capability of escaping local optima. The performance of ICEO is compared with 9 representative algorithms: CEO^16^, AO^23^, INFO^19^, GWO^24^, HSO^25^, TOC^26^, ESC^27^, HHO^28^, and PGA^29^.

#### Experimental setup

For all experiments, the dimensionality is set to $D=30$. Each algorithm is independently executed 30 times on each function. The maximum number of function evaluations (MaxFES) is set to 300, 000 as the termination criterion. For CEO and ICEO, the number of chaotic samples is set to 20. The theoretical global optimal values for F1–F7 are 0, while for F8 (Schwefel 2.26) the theoretical global optimum is $-12{,}569.487$. For benchmark functions whose theoretical global optimum is at the origin (e.g., Sphere and Schwefel 2.22), an optimal solution shift is implemented to relocate the global optimum to a non-zero coordinate to reduce potential bias caused by symmetry.

#### Results analysis

**Fig. 2:**
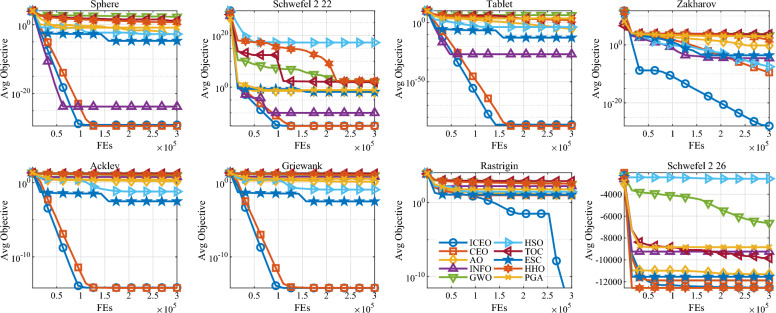
Convergence curves of 10 algorithms on 8 benchmark functions.

The performance of the 10 algorithms is evaluated across multiple indices, including solution accuracy, stability, convergence speed, and computational efficiency. The statistical results (Min, Mean, Max, Std, and Time) are summarized in Table 2, while the convergence process is visualized in Fig. 2. To further validate the statistical significance of the results, the Friedman test rankings are provided in Table 3.

As shown in Table 2, the proposed ICEO algorithm consistently achieves the highest solution accuracy across all 8 benchmark functions. For unimodal functions (F1–F4), ICEO and its predecessor CEO reach or come extremely close to the theoretical global optimum, significantly outperforming other meta-heuristic algorithms such as GWO, HSO, and TOC. Specifically, for F1, F2, F3, F7, and F8, ICEO achieves a minimum value, which can be attributed to the integration of the LS local search refinement and the self-learning Gaussian mutation. These mechanisms allow the algorithm to perform fine-grained exploitation around the current best solution, effectively overcoming the precision limitations of standard evolutionary operators. In terms of stability, ICEO exhibits the smallest standard deviation (Std) in most cases, indicating that the combined perturbation of Lévy flight and Gaussian mutation provides a robust balancing mechanism between exploration and exploitation, ensuring reliable performance across independent runs.

The convergence curves in Fig. 2 further highlight the efficiency of the ICEO’s search process. In the early stages, the chaotic initialization and enhanced search mechanisms enable ICEO to locate promising regions of the search space more rapidly than its competitors. For multimodal functions (F5–F8), while algorithms like HHO and ESC often suffer from premature convergence (as seen by their early plateaus), ICEO maintains a steady downward trend in objective values. This superior capability to escape local optima is primarily driven by the Lévy flight’s heavy-tailed jumps and the stagnation detection mechanism, which triggers the local search to refine the solution when evolution becomes stagnant. Regarding computational cost, although ICEO requires slightly more execution time than CEO and PGA due to the additional function evaluations consumed by the LS refinement and the more complex self-learning strategies, its runtime remains competitive with other high-performance algorithms like INFO and TOC. This marginal increase in computational overhead is well-justified by the significant gains in optimization precision and robustness.

The Friedman test results in Table 3 provide a comprehensive statistical confirmation of these observations. ICEO secures the 1st rank with an average Friedman rank of 1.45, followed by CEO with 2.06. Other algorithms such as ESC and INFO also perform well but fail to match the overall consistency of ICEO across different landscape types. The statistical evidence, combined with the detailed performance metrics, confirms that the proposed structural improvements in ICEO—specifically the self-learning perturbation and the adaptive local search—effectively enhance its search efficiency and robustness, making it a powerful tool for complex optimization problems.

**Table 2:** Statistical results of 10 algorithms on 8 benchmark functions ($D=30$).

Fcn	Metric	ICEO	CEO	AO	INFO	GWO	HSO	TOC	ESC	HHO	PGA
F1	Min	0	0	3.34e-21	7.89e-29	28.27	1.15e-08	0.21	5.95e-06	2.35	1.27e-28
	Mean	7.29e-30	3.62e-30	1.78e-03	1.53e-24	182.51	1.77e-03	13.68	1.92e-05	4.58	0.13
	Max	7.89e-29	1.58e-29	2.01e-02	4.52e-23	614.35	4.69e-03	183.44	3.67e-05	6.05	4.00
	Std	1.53e-29	5.23e-30	4.43e-03	8.25e-24	154.98	1.33e-03	33.77	7.11e-06	0.85	0.73
	Time	1.96	0.70	1.77	1.49	0.87	2.72	2.51	5.24	0.89	0.58
F2	Min	0	0	0	1.62e-14	32.53	3.61e-09	12.13	7.47e-03	199.52	0
	Mean	9.77e-16	8.59e-16	1.05e-02	1.00e-10	75.64	1.77e+17	77.03	1.33e-02	260.25	0.07
	Max	1.07e-14	7.11e-15	0.12	2.59e-09	186.59	5.05e+18	189.44	1.86e-02	356.59	1.00
	Std	2.31e-15	1.58e-15	2.54e-02	4.71e-10	37.63	9.21e+17	45.78	2.63e-03	37.54	0.25
	Time	1.99	0.64	1.33	1.50	0.81	2.77	2.43	5.31	0.85	0.53
F3	Min	0	0	3.62e-14	3.43e-83	3.84	1.03e-07	0.29	1.26e-14	34.47	1.04e-33
	Mean	5.65e-87	4.21e-88	7.33e-06	2.05e-27	1.81e+06	1.98e-04	506.77	2.01e-13	113.21	3.92e+03
	Max	1.29e-85	2.04e-87	7.03e-05	4.93e-26	3.58e+07	8.57e-04	5415.30	8.74e-13	306.52	1.18e+05
	Std	2.33e-86	8.17e-88	1.67e-05	9.20e-27	6.52e+06	2.54e-04	1133.17	2.19e-13	69.34	2.15e+04
	Time	3.05	0.91	2.41	1.82	1.20	3.53	2.78	6.95	2.00	0.90
F4	Min	3.37e-29	3.08e-12	4.34e-04	9.09e-13	287.91	3.97e-09	80.62	7.72e-05	29.93	10.39
	Mean	1.12e-28	3.10e-10	0.62	2.76e-05	1916.77	3.01e-08	5421.92	2.67e-04	55.21	1040.44
	Max	4.37e-28	3.36e-09	8.44	4.05e-04	4705.86	4.93e-07	14402.87	9.27e-04	92.45	4435.26
	Std	7.58e-29	6.37e-10	1.70	8.73e-05	970.98	8.80e-08	3930.72	1.63e-04	14.90	947.84
	Time	1.62	0.79	2.68	1.67	0.84	3.29	2.70	6.62	1.32	0.78
F5	Min	7.11e-15	3.55e-15	1.29e-10	1.34	2.76	2.36e-09	13.13	1.75e-03	3.16	4.26e-14
	Mean	7.34e-15	6.63e-15	1.58	5.78	8.67	6.33e-02	16.28	3.01e-03	11.94	2.62
	Max	1.42e-14	7.11e-15	11.02	19.32	13.04	1.18	19.96	4.17e-03	16.56	17.22
	Std	1.30e-15	1.23e-15	3.30	6.27	2.97	0.25	1.68	6.14e-04	5.95	4.61
	Time	3.61	0.67	1.99	1.69	0.80	3.13	2.45	6.45	1.01	0.77
F6	Min	3.55e-15	3.55e-15	1.83e-11	3.20e-14	2.54	1.38e-09	13.72	2.01e-03	2.87	4.26e-14
	Mean	6.51e-15	6.63e-15	1.38	6.40	8.52	0.11	16.04	2.96e-03	11.03	3.29
	Max	7.11e-15	7.11e-15	12.67	19.08	13.23	1.34	19.98	4.01e-03	16.82	17.97
	Std	1.35e-15	1.23e-15	3.59	6.44	3.07	0.35	1.35	5.59e-04	6.27	4.89
	Time	3.66	0.71	1.78	1.92	0.78	2.67	2.65	5.17	0.94	0.59
F7	Min	0	2.98	3.90	75.62	59.34	18.90	465.17	4.98	259.07	38.80
	Mean	0	10.18	13.41	181.25	318.75	31.54	851.41	12.08	392.19	66.58
	Max	0	20.89	175.82	372.11	995.18	53.73	1352.75	24.88	529.73	118.40
	Std	0	3.85	30.97	68.92	193.79	8.19	235.90	4.66	65.69	18.37
	Time	1.40	0.68	2.09	1.98	0.78	2.49	2.42	5.16	0.94	0.60
F8	Min	-12569.49	-12451.05	-11973.32	-10392.04	-7842.87	-4040.69	-12569.49	-12115.47	-12569.49	-10089.88
	Mean	-12537.90	-11873.33	-11313.32	-9244.34	-6635.37	-2581.83	-9844.30	-11547.32	-12569.49	-8836.76
	Max	-12332.61	-11266.66	-10434.56	-7713.20	-5459.42	-2047.80	-5403.32	-10909.83	-12569.48	-7247.30
	Std	69.08	311.55	364.55	631.29	643.19	512.98	1831.90	313.95	1.77e-03	640.13
	Time	3.38	0.76	2.04	2.16	0.81	2.51	2.60	5.46	0.98	0.70

**Table 3:** Friedman test ranking of 10 algorithms across 8 benchmark functions.

Fcn	ICEO	CEO	AO	INFO	GWO	HSO	TOC	ESC	HHO	PGA
F1	1.52	1.48	5.70	3.20	9.97	6.60	8.60	5.60	8.43	3.90
F2	2.03	2.17	4.78	4.07	7.73	8.63	7.73	5.93	9.70	2.22
F3	1.70	1.30	5.93	3.07	9.90	6.87	8.33	5.00	8.63	4.27
F4	1.00	2.07	6.00	3.50	9.07	3.47	9.53	4.97	7.10	8.30
F5	1.58	1.42	5.47	7.20	7.87	3.83	9.43	5.07	8.70	4.43
F6	1.48	1.52	5.33	7.23	7.70	3.83	9.47	5.00	8.40	5.03
F7	1.00	3.00	2.67	7.23	8.03	4.97	9.97	3.43	8.67	6.03
F8	1.27	3.57	4.90	7.00	8.97	10.00	5.92	4.23	1.78	7.37
Avg. Rank	1.45	2.06	5.10	5.31	8.65	6.03	8.62	4.90	7.68	5.19

## Application for WPS capacity configuration

### Case data and parameters

**Fig. 3:**
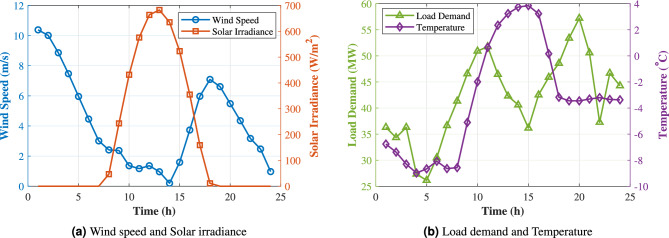
Typical daily meteorological and load data.

The typical daily profiles of wind speed, solar irradiance, load demand, and ambient temperature in the study area are illustrated in Fig. 3. Figure 3(a) shows the wind speed and solar irradiance, while Fig. 3(b) presents the load demand and temperature. In this case study, a wind–PV generation system is planned and coupled with an ESS to mitigate renewable intermittency and match supply with demand. The proposed optimization model is solved to determine the installed capacities of wind, PV, and storage by minimizing the total installed cost (including investment and O&M costs) subject to operational constraints and load-supply balance requirements.

Both economic and technical factors are considered in the capacity planning, including investment cost, O&M cost, and service life, as well as rated power, efficiency, and SOC limits. The technical parameters of the main equipment are listed in Table 4, and the corresponding economic parameters are provided in Table 5.

**Table 4:** Main equipment technical parameters.

Equipments	Parameters	Values
Wind components	Cut-in wind speed	3 m/s
	Rated wind speed	11.4 m/s
	Cut-out wind speed	24 m/s
PV modules	Open-circuit voltage	54.30 Voc/V
	Short-circuit current	15.60 Isc/A
	Temperature coefficient	-0.260%/$^\circ$C
Storage ESS	Charging efficiency	0.95
	Discharge efficiency	0.95
	SOC$_{\text {max}}$	0.8
	SOC$_{\text {min}}$	0.2

**Table 5:** Main equipment economic parameters.

Equipments	Investment cost (CNY/kW)	Operating cost (CNY/kWh)	Service life (year)
Wind power generation	2800	0.05	20
PV power generation	2400	0.03	20
Energy storage	2000	0.018	10

### Comparison of algorithms

**Fig. 4:**
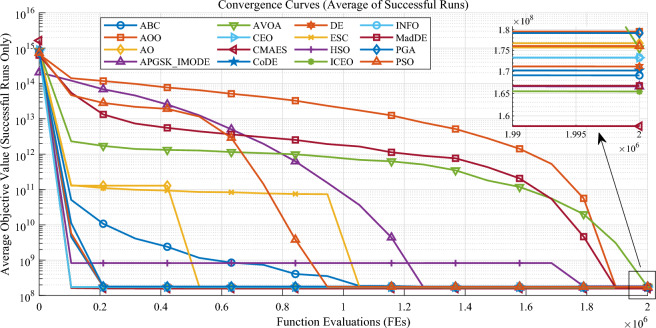
Comparison of average objective convergence curves of different algorithms over successful runs only.

To comprehensively evaluate the effectiveness of the proposed ICEO for the WPS capacity configuration problem, it is compared with 20 representative meta-heuristic algorithms that cover different generations of optimization methods. Specifically, the comparison set includes several widely used classical algorithms, namely DE^30^, PSO^18^, CMAES^31^, CoDE^32^, ABC^33^, and CLPSO^34^. It also includes a group of popular algorithms developed in recent years and/or widely adopted in the community, such as CEO^16^, AO^23^, INFO^19^, GWO, HSO, TOC, AOO^35^, AVOA^36^, ESC^27^, HHO, PGA, and RUN^37^. Among them, GWO has been cited more than 20,000 times and HHO has been cited more than 6,000 times, indicating their broad influence and practical relevance. In addition, APGSK_IMODE^38^ and MadDE^39^ are two competitive algorithms that achieved top performance in CEC benchmark competitions, where APGSK_IMODE ranked top1 in CEC2021 and MadDE ranked top2 in CEC2021. For a fair comparison, all algorithms use their default parameter settings; the population size is set to 50 and the maximum number of function evaluations is set to $2 \times 10^6$ for all algorithms. For CEO and ICEO, the number of chaotic samples is set to 20. Each algorithm is independently executed 30 times. The convergence behaviors are shown in Fig. 4, and the statistical performance indicators are summarized in Table 6.

Figure 4 presents the average convergence curves of the objective function over successful runs only. Overall, ICEO converges faster than most comparison algorithms and reaches a competitive convergence accuracy, with its average performance being second only to CMAES. In contrast, ESC, AO, and HSO are prone to stagnation and may be trapped at local optima, which limits further improvement in the later stage. Meanwhile, AVOA, AOO, MadDE, and APGSK_IMODE exhibit relatively slower convergence, requiring more evaluations to approach their final objective values. These observations indicate that ICEO achieves a better balance between convergence speed and solution quality for the WPS capacity configuration problem.

To demonstrate the differences in performance among various algorithms during the optimization process, key indicators of the optimization results for each algorithm are summarized, and the relevant statistical data are shown in Table 6. Table 6 reports the statistical comparison results over 30 independent runs. ICEO achieves the lowest cost (151,805,080 CNY) among all tested algorithms, outperforming the strongest competing algorithm in terms of best cost (CMAES with 154,126,997 CNY). Moreover, ICEO maintains a 100% success rate while keeping a competitive runtime (7.90 s), demonstrating both robustness and computational efficiency. Among the classical algorithms, DE, PSO, CMAES, CoDE, and ABC generally achieve feasible solutions, whereas CLPSO fails to find feasible solutions in this case (0% success rate). Among the popular and recent algorithms, several methods (e.g., GWO and HHO) also show 0% success rate under the same settings, indicating that feasibility handling is a critical challenge for this complex constrained planning task. Overall, the results in Fig. 4 and Table 6 consistently verify that ICEO provides a superior balance between solution quality, stability, and feasibility for the WPS capacity configuration problem.

**Table 6:** Algorithm performance comparison.

Algorithms	Best cost (CNY)	Average cost (CNY)	Worst Cost (CNY)	Std Dev (CNY)	Runtime (s)	Success rate (%)
ABC	159949443	169101509	186816567	7102223	62.64	100%
AOO	163113072	179548543	205815564	10860677	9.14	90%
AO	163492522	176698036	201577807	9436304	15.51	100%
APGSK_IMODE	156508350	166570513	176059520	5443919	134.17	100%
AVOA	163680364	175449421	200753748	8565547	6.42	87%
CEO	161676069	173254168	202549689	7263235	5.50	100%
CLPSO	—	—	—	—	6.02	0%
CMAES	154126997	157768342	165369369	2559292	30.84	63%
CoDE	158363411	170247099	180928573	5454825	6.00	100%
DE	157736032	171179526	184795967	6968284	4.39	100%
ESC	164189416	175675658	194775237	8469936	25.12	100%
GWO	—	—	—	—	5.02	0%
HHO	—	—	—	—	10.55	0%
HSO	164127725	182740799	223715110	14000570	16.74	100%
INFO	158725912	173274986	188286842	8031712	9.57	100%
MadDE	166583390	166727950	166914309	169373	5.73	10%
PGA	160855540	179169221	207196296	12808140	5.40	100%
PSO	162601335	176019613	191599881	7934834	7.77	100%
RUN	—	—	—	—	8.55	0%
TOC	—	—	—	—	14.13	0%
**ICEO**	**151805080**	**165427881**	**184982728**	**7642689**	**7.90**	**100%**

**Fig. 5:**
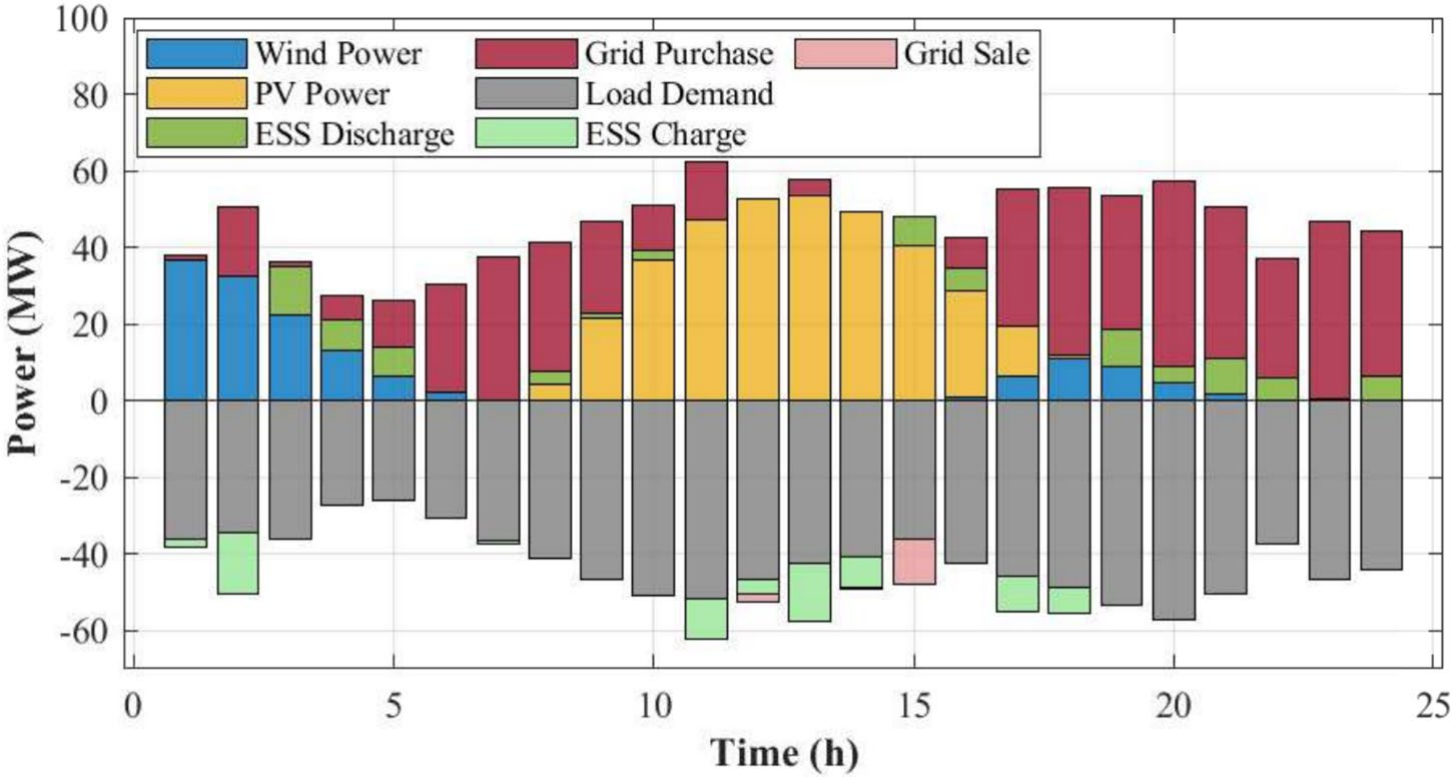
Optimal daily scheduling power obtained by ICEO.

Based on the ICEO solution, the configured capacities are 48.62 MW for wind power, 50 MW for PV, and 65 MWh for ESS. The corresponding optimal daily scheduling results are presented in Fig. 5. Figure 5 presents the optimal daily power scheduling results obtained by ICEO. During periods of high solar irradiance, PV generation supplies a large share of the load and any surplus is absorbed by the ESS through charging. During nighttime hours with higher wind speeds, wind generation becomes the dominant contributor and complements PV generation, thereby reducing the required discharge from the ESS. When the aggregated renewable output falls below the load demand, the ESS discharges to maintain power balance; conversely, surplus renewable power is stored via charging to support subsequent deficit periods.

**Fig. 6:**
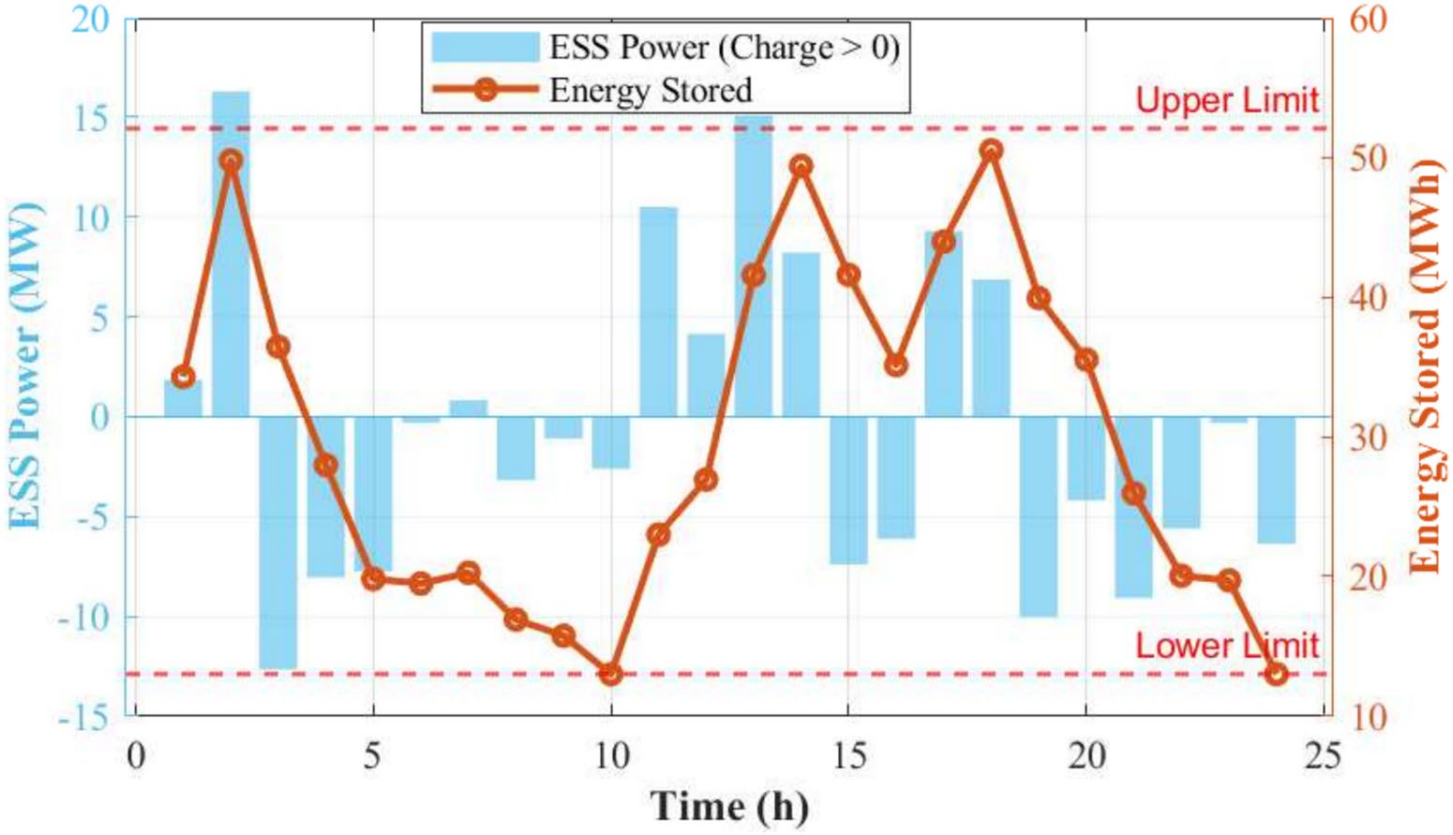
Power and energy state (SOC) of the ESS.

Figure 6 illustrates the power and energy state (SOC) of the ESS throughout the typical day. The results show that the ESS power output and SOC are within the prescribed limits, ensuring the safe and reliable operation of the system. During periods of surplus renewable energy, the ESS charges to store energy, causing the SOC to increase. Conversely, when renewable energy generation is insufficient to meet the load, the ESS discharges, leading to a decrease in SOC. The flexible operation of the ESS effectively buffers the stochasticity of wind and solar power, enhancing the system’s ability to maintain power balance.

## Conclusion

This study investigates the capacity optimization of a WPS hybrid system. A multi-energy complementary system architecture and an energy management formulation capturing multi-source coupling are established. The capacity planning problem is formulated to minimize the total installed cost subject to operational constraints. An improved chaotic evolution optimization algorithm (ICEO) is then developed and applied to solve the resulting constrained optimization problem. Three main conclusions are drawn.

(1) ICEO integrates Gaussian mutation, Lévy-flight-based perturbation, and stagnation-triggered local search, which jointly improves the convergence stability and solution accuracy compared with the original CEO algorithm.

(2) Benchmark tests on eight standard functions demonstrate that ICEO consistently achieves competitive solution accuracy and stability compared with nine representative optimizers, confirming the effectiveness of the proposed self-learning perturbation and stagnation-triggered local search in improving search efficiency.

(3) In the WPS case study, ICEO yields a feasible capacity configuration (48.62 MW wind, 50 MW PV, and 65 MWh ESS) with a lower total installed cost than competing algorithms, while satisfying operational constraints such as power balance and SOC limits.

## Data Availability

The raw data supporting the findings of this study are available from the corresponding author at any time upon request.
